# Transcriptome sequencing and analysis of major genes involved in calcium signaling pathways in pear plants (*Pyrus calleryana* Decne.)

**DOI:** 10.1186/s12864-015-1887-4

**Published:** 2015-09-30

**Authors:** Yuanyuan Xu, Xiaogang Li, Jing Lin, Zhonghua Wang, Qingsong Yang, Youhong Chang

**Affiliations:** Jiangsu Academy of Agricultural Sciences; Jiangsu Key Laboratory for Horticultural Crop Genetic Improvement, Institute of Horticulture, Nanjing, 210014 People’s Republic of China

**Keywords:** Calcium signaling pathways, Pear (*Pyrus calleryana* Decne.), RNA-Seq, Salt stress, Transcriptome

## Abstract

**Background:**

Pears (*Pyrus* spp. L.) are an important genus of trees that produce one of the world’s oldest fruit crops. Salinity stress is a common limiting factor for plant productivity that significantly affects the flavor and nutritional quality of pear fruits. Much research has shown that calcium signaling pathways, mediated by Calcineurin B-like proteins (CBLs) and their interacting kinases (CIPKs), are closely associated with responses to stresses, including salt. However, little is known about the molecular mechanisms that govern the relationship between salt stress and calcium signaling pathways in pear plants. The available genomic information for pears has promoted much functional genomic analysis and molecular breeding of the genus. This provided an ample foundation for characterizing the transcriptome of pear under salt stress.

**Results:**

A high-throughput Illumina RNA-seq technology was used to identify a total of 78,695 unigenes that were successfully annotated by BLASTX analysis, using the publicly available protein database. Additionally, 2,855 novel transcripts, 218,167 SNPs, 23,248 indels and 18,322 alternative splicing events occurred. Assembled unique sequences were annotated and classified with Gene Ontology (GO), Clusters of Orthologous Group (COG) and Kyoto Encyclopedia of Genes and Genomes (KEGG) analysis, which revealed that the main activated genes in pear are predominately involved in functions such as basic physiological processes, metabolic pathways, operation of cellular components, signal transduction mechanisms, and other molecular activities. Through targeted searches of the annotations, the majority of the genes involved in calcium signaling pathways were identified, among which, four genes were validated by molecular cloning, while 11 were validated by RT-qPCR expression profiles under salt stress treatment.

**Conclusions:**

These results facilitate a better understanding of the molecular genetics and functional genomic mechanisms of salt stress in pear plants. Furthermore, they provide a valuable foundation for additional research on the molecular biology and functional genomics of pear and related species.

**Electronic supplementary material:**

The online version of this article (doi:10.1186/s12864-015-1887-4) contains supplementary material, which is available to authorized users.

## Background

Soil salinization is a severe problem encountered worldwide in agricultural production that causes abiotic stress to plants and lowers crop growth and yields, thus impacting food security [1]. The accumulation of sodium in soils ultimately leads to high concentrations of sodium ions (Na^+^) within plant cells [2]. Primary sources of salinity include natural processes such as mineral weathering or fluctuation of sea water levels, along with anthropogenic activities such as irrigation [3]. An overload of salinity in plants is known to cause a series of negative effects including reduction in water availability, mineral imbalances, and ultimately altered seed germination and plant metabolism [4]. Given the world’s accelerating population growth and corresponding pressure on food supplies, this serious problem demands global solutions, such as improvement of the salt tolerance of crops [5]. Thus, elucidating the molecular basis of salt-stress signal transduction pathways and salt tolerance mechanisms is fundamental to understanding the biology of salt-tolerant plants, in order to support strategies for the design, genetic engineering and breeding of salt-tolerant crops [6].

Plants have evolved complex signaling pathways in response to salt-stress, and have acquired plasticity in metabolic functions and developmental switches to cope with such changing environmental conditions [6–8]. Plant responses to signals are encoded by different Ca^2+^ signatures. The major family of Ca^2+^ sensors includes calmodulins (CaMs), calcineurin B-like proteins (CBLs), calcium-dependent protein kinases (CDPKs/CPKs) and CBL interacting protein kinases (CIPKs). These sensors contribute to intricate signaling networks in plants, and they are encoded by complex families of genes [9, 10]. Considerable recent attention has been directed at explaining the molecular basis of plant salt tolerance. Most studies have emphasized the significance of Ca^2+^ signals in reinstating cellular ion homeostasis, demonstrating the importance of the calcium sensor and signaling pathways involved in salt stress signal transduction such as the CBL-CIPK and CDPK pathways that were identified in *Arabidopsis* and rice [11–13]. Research has shown that different, interconnected pathways may jointly coordinate regulation of plant responses to salt stress [6, 8].

Pear trees (*Pyrus* spp. L.) are cultivated widely throughout the world. They are the third most important fruit crop in temperate zones, after grape and apple [14, 15]. The genome of this genus in the family Rosaceae (subfamily Pomoideae) was published recently; it contains 42,812 genes, providing a rich resource for research by plant molecular biologists [15]. In recent years, the production of pears in Southeast coast of China is reportedly quite limited due to further spread of soil salinization [16]. Considerable effort has been directed at investigating salt stress in pear plants, especially its accumulation, translocation, and physiological and metabolic effects [17, 18]. However, little is known about the mechanisms underlying the responses of pear plants to salt stress at the molecular level, especially regarding relationships among the key genes that control salt stress and calcium signaling pathways.

Following release of the pear genome sequence, several studies have reported functional genomics research based on the analysis of transcription-level data. For example, RNA-seq technology has been used to identify genes related to bud dormancy in *P. pyrifolia*, and to study transcriptome profiling of fruit development and maturation in *P. bretschneideri* ‘Rehd’ (Chinese white pear) [19–21]. Despite this obvious potential, to date, RNA-seq analyses of the genome-wide responses of major genes involved in calcium signaling pathways under salt stress have not been assessed in pears.

Our objective was to use the RNA-seq technology platform to characterize the transcriptome of pear seedlings that were subjected to salt stress. A large set of transcript sequences for pear was obtained to identify the majority of activated genes involved in this process. The candidate genes involved in calcium signaling pathways were identified successfully, and the sequence of representative genes and expression patterns were further validated. The leaves *de novo* transcriptome based on RNA-seq was comprehensively characterized in pear plants. This would provide a publicly available foundation of information to facilitate both an improved understanding of the molecular mechanisms that regulate calcium signaling pathways in plants under salt stress, and the genetic improvement of traits governing fruit quality in molecular breeding programs for pear plants.

## Results and discussion

### Annotation of predicted proteins

Among 95,727 unique sequences that we identified, 78,695 of them (82.21 %) significantly matched a sequence in at least one of the five public databases that we consulted (Table 1). The rate of annotated unigenes was higher than the range of previous studies in other species (58 % in safflower flowers and 58.01 % in Chinese fir), indicating the relative integrity and conserved functions of the assembled transcripts sequences in pear [22, 23]. The size-distribution of the predicted proteins and coding sequence (CDS) are shown in Additional file 1A and B, respectively. ESTScan was used to predict the coding regions of the remaining 17.79 % of the unique sequences (17,032) that did not match any in the databases. An additional 1,824 unique sequences (1.91 %) also showed orientation in the coding sequence (Additional file 1C and D). The absence of homologous sequences in public databases may indicate that novel genes are specifically expressed in pear leaves, or, it may be due to other biological or technical biases such as assembly parameters. Moreover, the reason why some cDNAs are non-coding or highly variable needs to be further explored [24, 25].

**Table 1:** Summary statistics of functional annotation for pear unigenes in public databases

Public protein database	No. of unigene hits	Percentage
NR	72,616	75.86
NT	70,691	73.85
SwissProt	41,255	43.10
KEGG	36,179	37.79
COG	16,915	17.67
GO	54,279	56.70
ALL	78,695	82.21

**Fig. 1:**
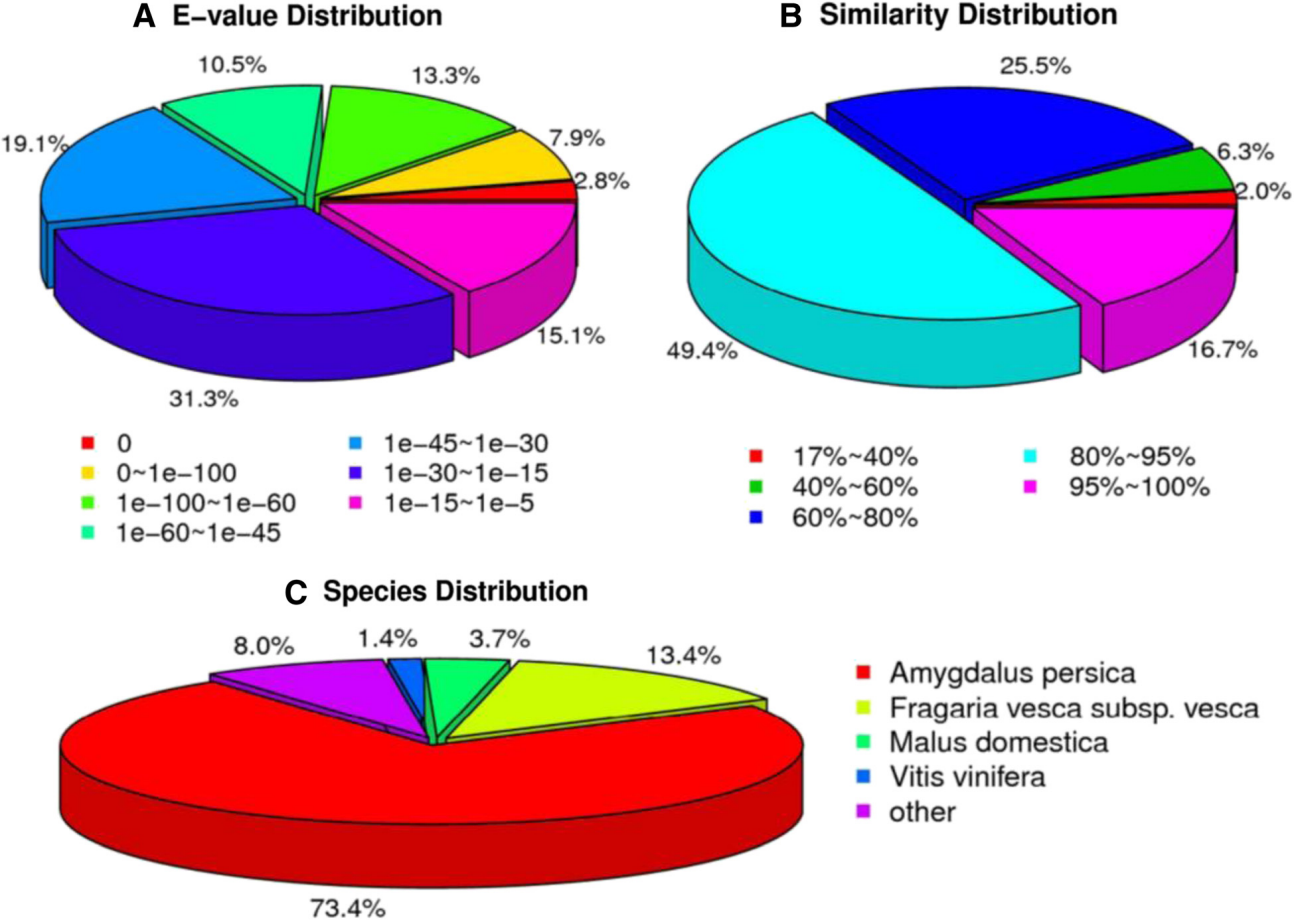
Characteristics of homology search for Illumina sequences against the NR database. **a** E-value distribution of BLAST hits for matched unique sequences with an E-value cut-off of 1.0E^−5^. **b** Similarity distribution of the top BLAST hits for each unique sequence. **c** Species distribution is shown as the percentage of the total homologous sequences (with an E-value ≤1.0e^−5^. All plant proteins in the NCBI NR database were used in the homology search and the best hits of the sequences were extracted for analysis

The E-value distribution of the top hits in the NR database showed that 34.42 % of the mapped sequences had strong homology with the E-value <1.0E^−45^, whereas 65.57 % of the homolog sequences ranged from 1.0E^−5^ to 1.0E^−45^(Fig. 1a). The distribution of similarity values showed that 66.1 % of the query sequences had a similarity of more than 80 %, while 33.8 % of the hits had a similarity ranging from 17 % to 80 % (Fig. 1b). In terms of species distribution, the majority of the annotated sequences corresponded to known nucleotide sequences of plant species, with 73.4 % of the sequences showing the highest homology to sequences from *Amygdalus persica*, followed by *Fragaria vesca* subsp. *vesca* (13.4 %), *Malus domestica* (3.7 %) and *Vitis vinifera* (1.4 %) (Fig. 1c). The two species with the most BLAST hits belonged to the *Rosaceae* family, indicating that the sequences of the pear transcripts obtained in the present study were annotated properly [26].

### Functional annotation and classification of the assembled unique sequences

**Fig. 2:**
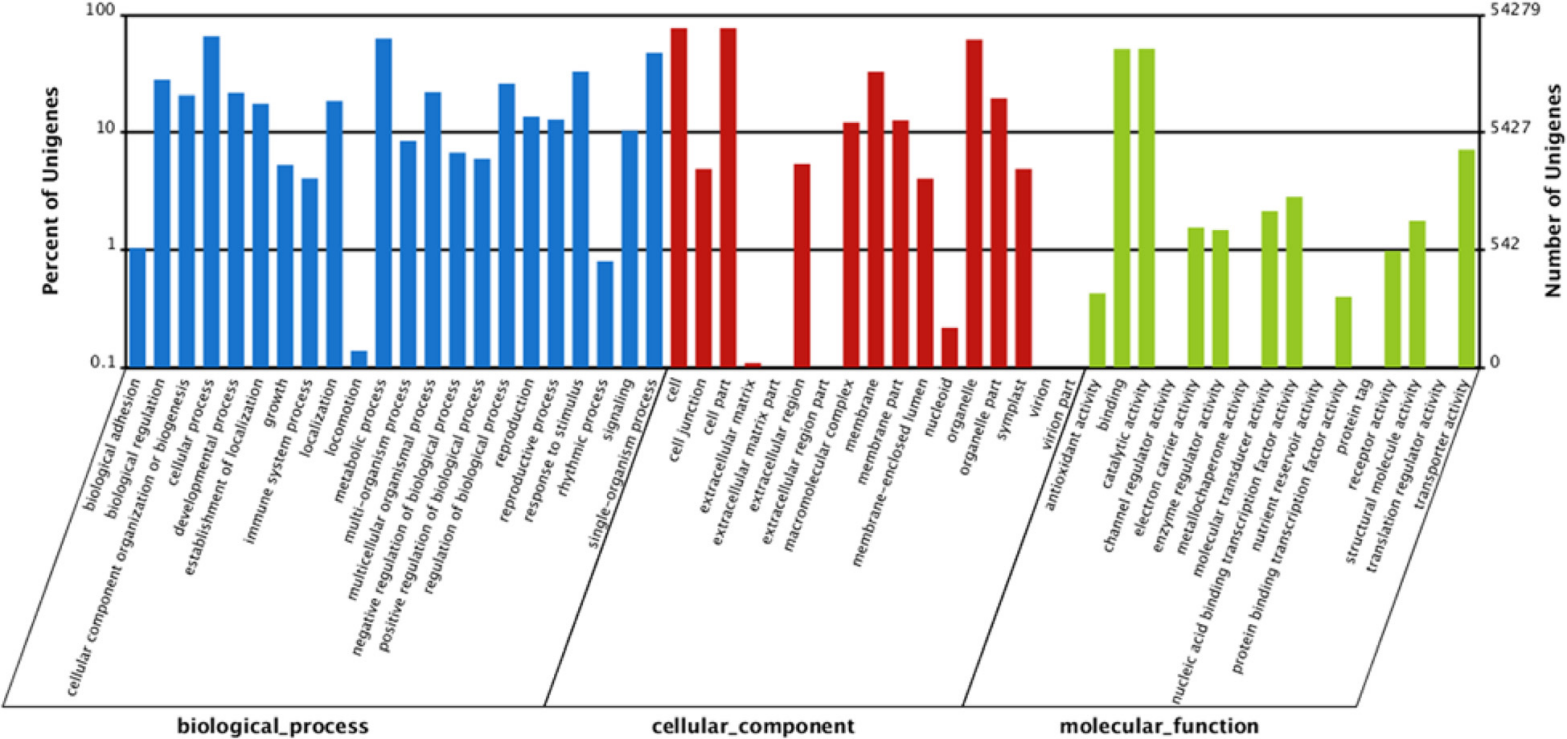
Histogram presentation of Gene Ontology classification. The results are summarized as three main categories: biological processes, cellular components and molecular functions

**Fig. 3:**
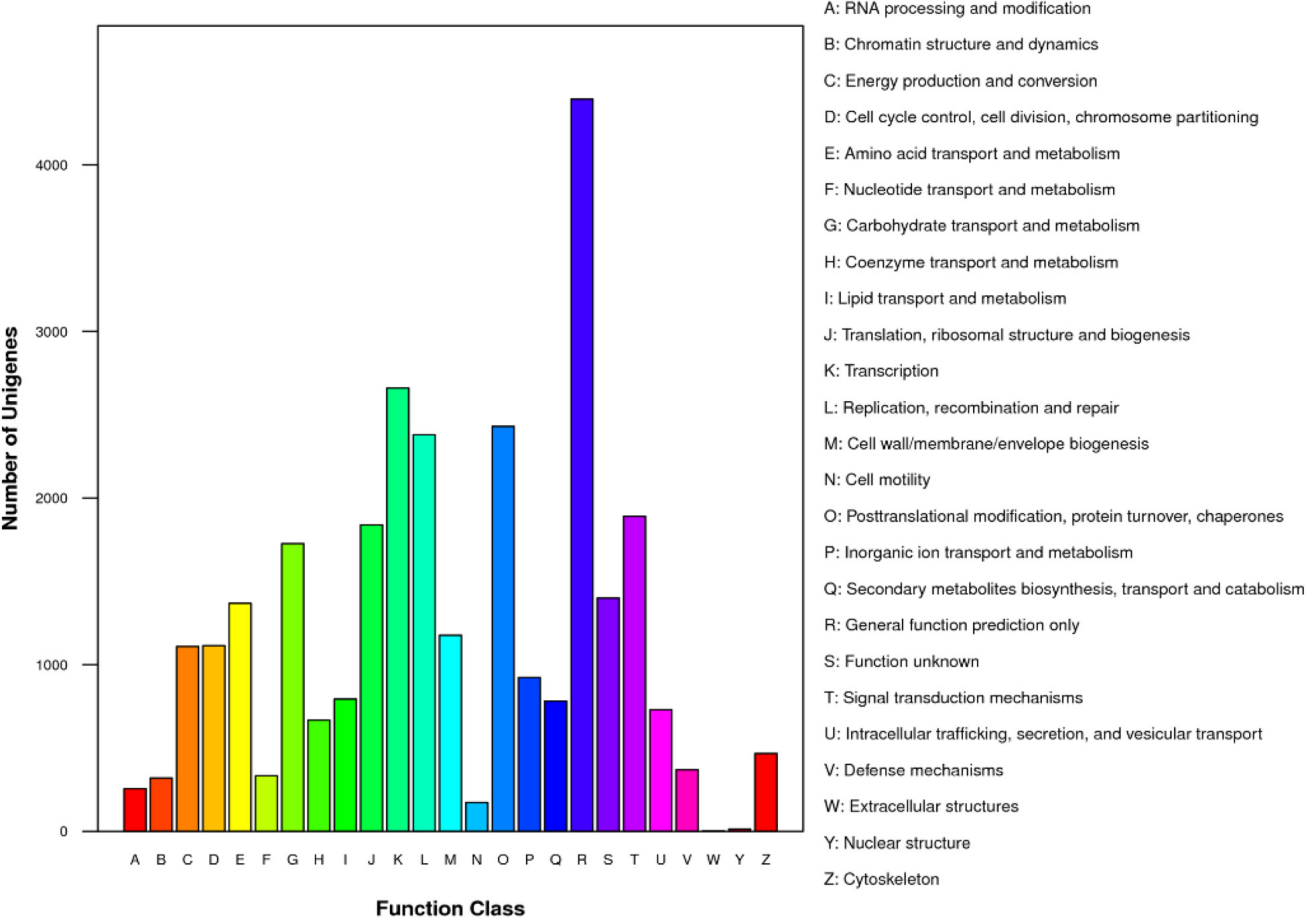
COG function classification of the PDX transcriptome

GO assignments can provide standardized vocabulary for assigning functions of the uncharacterized sequences and hence were used to classify the functions of the predicted genes in pear [27]. Based on sequence homology, 54,279 sequences were categorized into 55 functional groups, including biological processes, cellular components, and molecular functions (Fig. 2). Within each of the three main categories of GO classification, the dominant subcategories were “cellular process” (35,718) among biological processes, “cell” (41,752) among cellular components, and “catalytic activity” (27,825) among molecular functions. That is, the major GO classifications involved in the annotated sequences controlled basic biological regulation and metabolism. These findings were consistent with previous studies of transcriptome analysis in radish roots and sweet potato tuberous roots [24, 28]. COG classification was used to further evaluate the effectiveness of the annotation process and the completeness of the transcriptome library. A COG database was built from classifications of phylogenetic relationships, consisting of protein sequences encoded in 21 complete genomes including those of bacteria, eukaryotes and algae [29]. Each COG classification consists of groups of paralogs or individual proteins from at least three lineages, and thus corresponds to an ancient conserved domain [25]. In total, 16,915 of 95,727 (17.67 %) sequences were assigned to the COG classification (Table 1). As some of these sequences were annotated with multiple COG functions, altogether 29,337 functional annotations were generated. Among the 25 COG categories, the cluster for “General function prediction only” (4,395, 14.98 %) represented the largest group, followed by “Transcription” (2,662, 9.07 %), “Posttranslational modification, protein turnover, chaperones” (2,431, 8.29 %) and “Replication, recombination and repair” (2,381, 8.12 %). The categories of “extracellular structures” and “nuclear structures” were the smallest groups (Fig. 3).

**Fig. 4:**
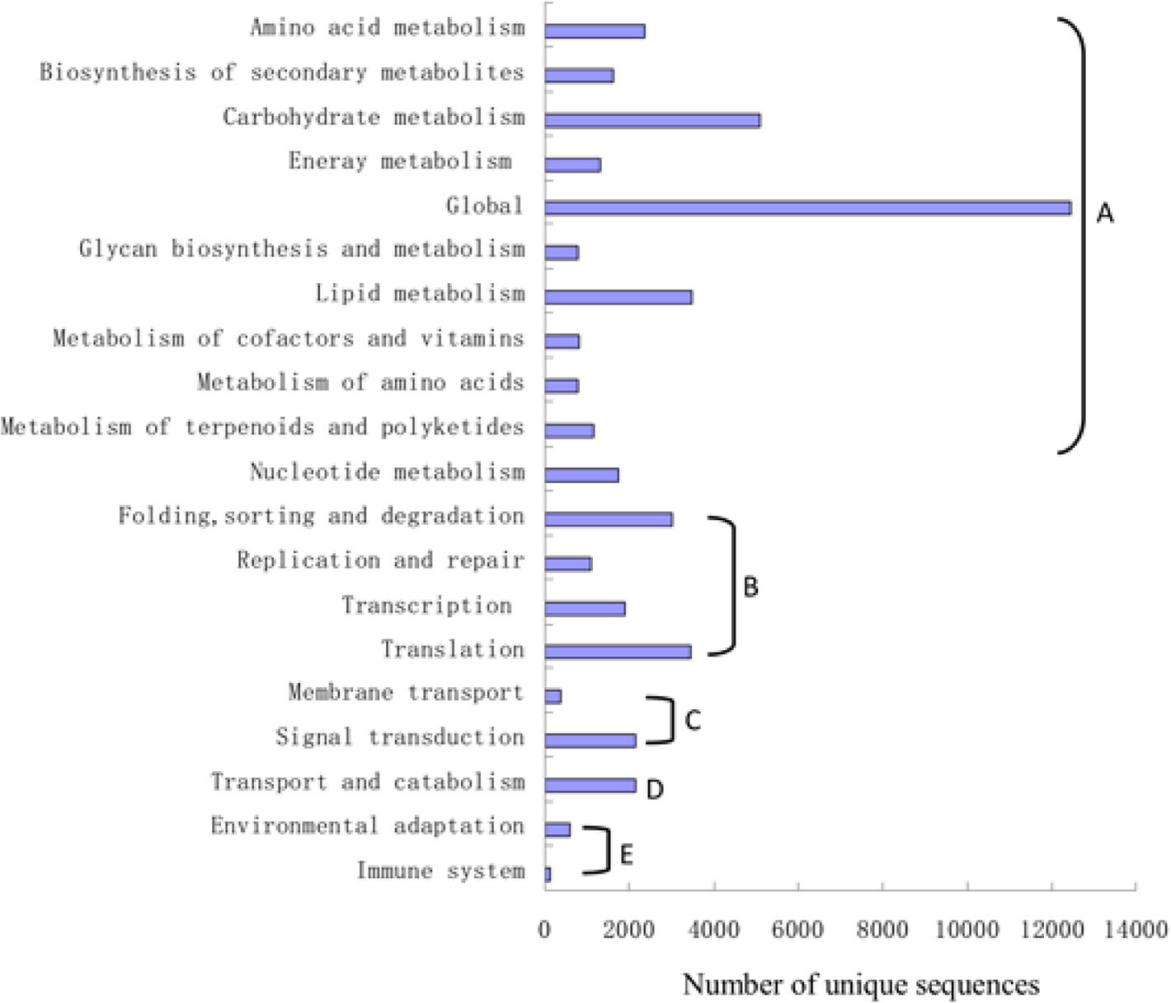
Pathway assignment based on KEGG. Pathways were assigned into five categories. A: Metabolism; B: Genetic information processing; C: Environment information processing; D: Cellular processes; E: Organismal systems

The KEGG pathway database can facilitate a systematic understanding of the molecular interactions among genes, in terms of networks [30]. Pathway-based analysis enables further understanding of those biological functions. In order to identify the biological pathways active in pear, the unique sequences were annotated with KEGG Orthology (KO) numbers from BLASTX alignments against the KEGG database, using a cut-off E value of 10^−5^. A total of 36,179 (37.79 %) annotated sequences were matched in the KEGG database and were assigned to 128 active pathways. The five largest pathway groups were metabolic pathways [ko01100, 8435, 391 (23.31 %)], biosynthesis of secondary metabolites [ko01110, 4,000 (11.06 %)], plant-pathogen interactions [ko04626, 1,904 (5.26 %)], plant hormone signal transduction [ko04075, 1,814 (5.01 %)] and spliceosomes [ko03040, 1,239 (3.42 %)] (Additional file 2). Figure 4 shows the features of the pathway assignment based on KEGG. The majority of the assigned unique sequences coded for related genes involved in primary metabolites, such as carbohydrate metabolism, lipid metabolism and amino acid metabolism. However, only 1,606 unique sequences that coded for the genes were associated with secondary metabolites. These annotations of protein or gene descriptions, putative conserved domains, and potential metabolic pathways will provide a valuable resource for subsequent investigation of specific processes, structures, biological functions and pathways in pear growth and development.

### Identification of novel transcripts, SNPs, indel and alternative splicing events

**Fig. 5:**
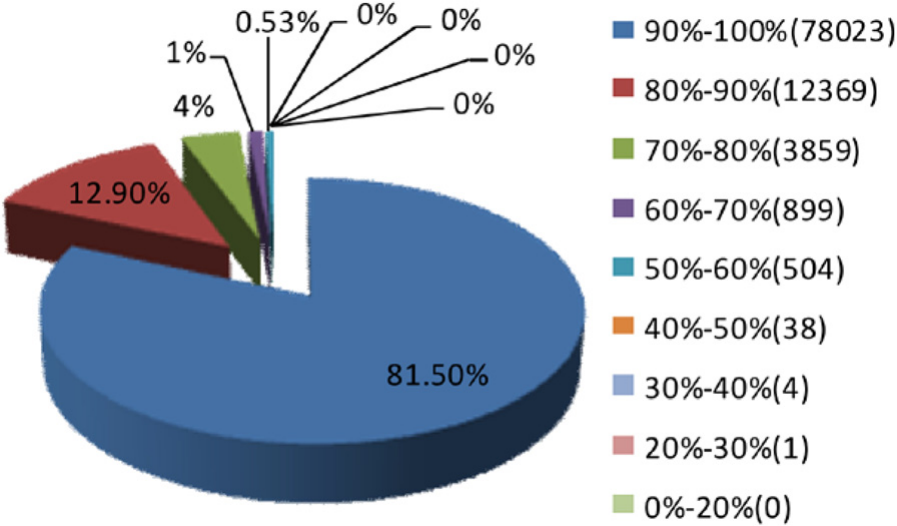
Gene coverage distribution of PDX. Pie sections with different colors represent proportions of genes with certain coverage

**Fig. 6:**
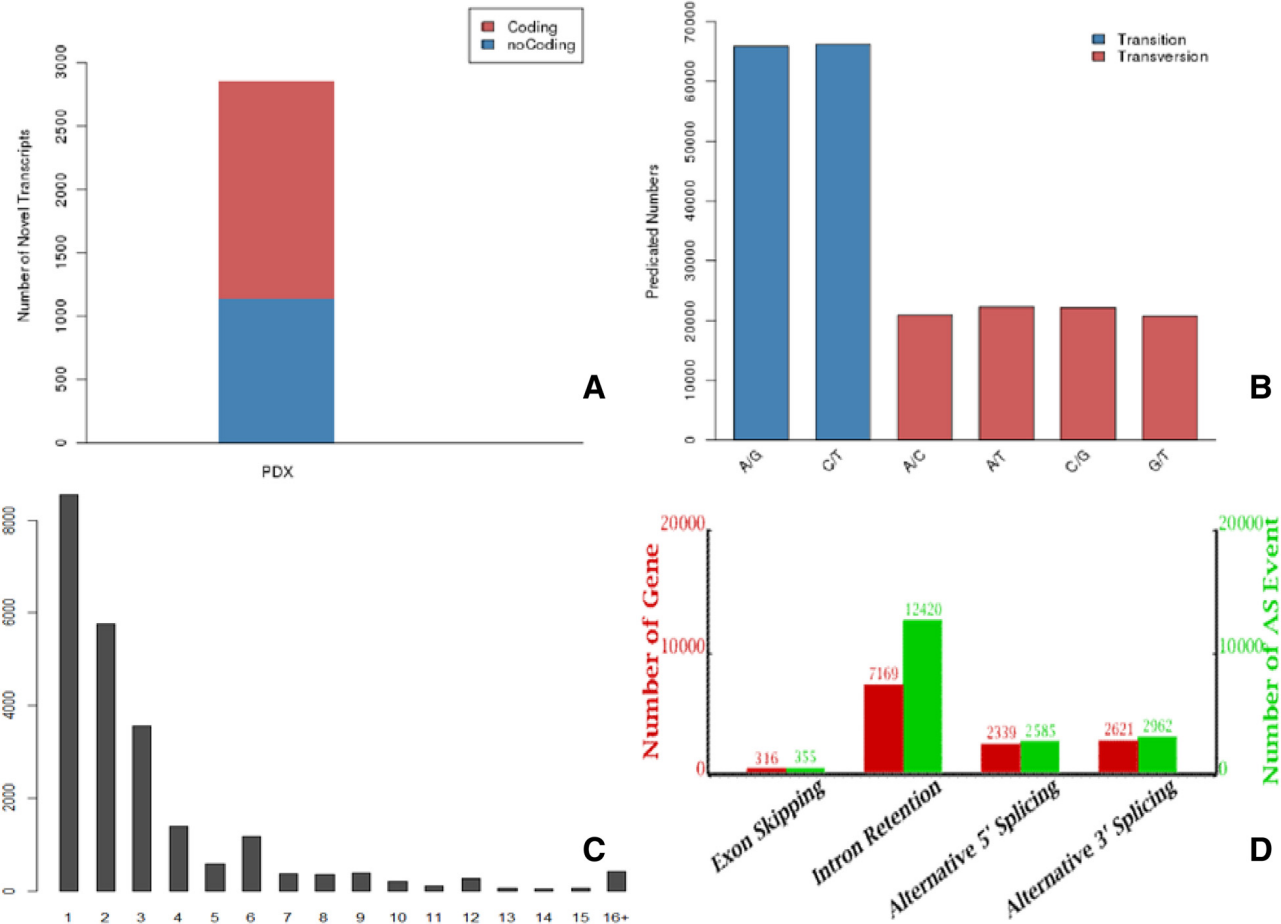
Discovery of novel transcripts (**a**), SNP types (**b**), indels (**c**) and alternative splicing (**d**) in the unique sequence assembled and aligned with the pear genome.

During the RNA-Seq experiment, mRNA-enriched RNAs were firstly broken into short segments by chemical methods and then sequenced. Read alignment to the reference genome was performed using the TopHat/Cufflinks/Bowtie/SOAP and Coding Potential Calculator [31]. Gene coverage was calculated as the percentage of a gene covered by reads. This value is equal to the ratio of the base number in a gene covered by unique mapping reads to the total number of bases in the coding region of that gene [32, 33]. A total of 78,023 (82 %) unique sequences with coverage between 90 %–100 % became the largest group, whereas no unique sequences were identified with coverage between 0 %–20 % (Fig. 5). After demultiplexing, merging and filtering of reads, about 26.8 million clean reads were obtained in the PDX sample of our experiment; 64.58 % of the unique transcripts were sequenced and successfully mapped to the pear genome, and 45.39 % were mapped to the *de novo* assembled results of pear (Table 2). As previously reported, an unprecedentedly high number of novel transcripts have been discovered in the genomes of humans, mice, and many plant species [31, 34]. In our research, a total of 2,855 transcripts were presumably displaying potentially novel isoforms for all the transcripts (Additional file 3). Additionally, results from Coding Potential Calculator analysis revealed that 1,720 potentially novel transcripts had the ability to code proteins, but the specific functions of these proteins remain to be determined (Fig. 6a). In order to provide the best genome coverage for the analysis of performance and production traits, numerous SNPs are needed [35, 36]. SNPs include transition and transversion of the nucleotide; in this study, among the 218,167 SNPs found, 60.5 % (132,070) were transitions between ‘A/G’ and ‘C/T’, whereas 39.5 % (86,097) were transversions among ‘A/C, A/T, C/G and G/T’ (Fig. 6b). The SNPs identified in this report provide a highly useful baseline for further genetic studies in pear, and will contribute to the development of a high density SNP array.

**Table 2:** Summary statistics for output sequencing

Sample name	Clean reads	Genome map rate	Gene map rate	Expressed genes	Novel transcripts	Alternative splicing events	SNPs
*PDX*	26,804,620	64.58 %	45.39 %	95,697	2,855	18,322	218,167

In addition, 23,248 insertion/deletion (indels) were identified, including 10,366 insertions and 12,881 deletions, with a homozygous:heterozygous ratio of 0.8. Of the total, the proportion of single-base indels was highest (8,545, 36.8 %), followed by double-base (5,755, 24.8 %) and triple-base indels (3,555, 15.3 %). The lengths of many indels were integral multiples of three bases, according to the indel length distribution (Fig. 6c). The indels were annotated according to the gene annotation information of birch-leaf pears. More than half of the indels were located in genic regions (13,212, 56.8 %); another 10,036 were intergenic (43.2 %). Among total 23,248 indels, 9,500 (40.8 %) were within regions 2 kb upstream or downstream of genes, while 536 indels were more than 2 kb away from genes. Furthermore, 6,110 (26.3 %), 4,818 (20.7 %) and 2,269 (9.8 %) indels among total 23,248 indels were located within CDS, UTR and introns respectively, and 3,320 (14.3 %) indels led to frameshifts (Additional file 4).

The splicing of precursor mRNA not only reconstitutes gene coding sequences, but it is also an important regulatory step in the process of gene expression in plants [37, 38]. Alternative splicing is a major means of increasing the transcriptome and proteome diversity in plants or mammals for a given number of genes [39, 40]. RNA-seq provides an efficient opportunity to investigate alternative splicing, which has revealed numerous instances of alternative splicing in mammals and plants [32, 41, 42]. We found evidence of 18,322 alternative splicing events, and IR was the major splicing pattern detected (67.8 % of total events), while ES was rare (only 1.9 % of total events). The number of genes involved in the four types of alternative splicing were 316 for ES, 7,169 for IR, 2,339 for A5SS, and 2,621 for A3SS (Fig. 6d). These alignment results and analysis represented a significant improvement over the previous genome annotation and enabled us to further investigate gene expression profiles.

### Identification of genes involved in MYB transcription factors

MYB transcription factors have been implicated in controlling cell development by responding to varieties of biotic and abiotic stresses, and by being involved in signal transduction processes of plant growth, including calcium signaling pathways [43, 44]. It has also been reported that promoters of related genes in calcium signaling pathways usually contain the same cis-acting elements, indicating that these promoters can be regulated by the same transcription factors [45]. The expression of the genes in calcium signaling pathways is regulated by the transcription regulation gene, thereby significantly improving the resistance of plants to salt stress. For example, MYB1 in *Boea crassifolia*, MYB4 in *Oryza sativa* and MYB2 in *Chrysanthemum* spp. showed a response to salt stress [46–48]. Soybean MYB transcription factors were also identified as being part of the stress tolerance apparatus based on salt stress assays in transgenic plants [49]. From the pear transcriptome analysis in this study, a total of 633 unique sequences were predicted to code MYB proteins including a large number of members (e.g., MYB1, 6, 15, 17, 32, 44, 58, 78, 107, 108, 109, 112, 113, 114, 115, 116, 118; Additional file 5). However, the specific function of each particular MYB member in calcium signaling pathways or salt-stress responses in pear needs to be verified using a functional genomics approach.

### Identification of genes involved in the calcium signaling pathways of pear

Previous studies suggest that Ca^2+^ plays an important role in the signal transduction pathway of eukaryotes [6, 7]. In plants, intracellular calcium levels are altered in response to various environmental stresses, including high concentrations of salts [8]. An additional level of regulation in calcium signaling is achieved via the action of calcium-binding proteins, including CDPKs, CaMs, CMLs and CBLs [9]. Based on the genes currently known to be involved in calcium signaling pathways in *A. thaliana* and rice, 75 unigenes in our transcriptome dataset were found to be homologous to the previously identified genes encoding calcium-binding proteins. The results indicated that those genes control responses to salt stress signals, and that the function is relatively conserved among different species. Moreover, four unigenes were found to be homologous to the genes encoding calcineurin B-like protein (Additional file 6), which plays a critical role in responses to salt stress. In most cases, more than one unique sequence was annotated as encoding the same protein; such sequences may represent different members of a gene family and/or fragments of a single transcript [50].

*CaMs* play important roles in the regulation of growth, development and abiotic stress resistance and they have been identified in several plant species [51–53]. The *CML* family is likely to have evolved from *CaMs*. Genome-wide identification of *A. thaliana* and rice has revealed that *CaM* and *CML* proteins are typically encoded by gene families. The *A. thaliana* genome harbors 7 *CaM* and 50 *CML* genes that encode potential calcium sensors [51]. In our annotated pear transcriptome unigene dataset, 32 sequences were successfully identified that corresponded to seven homologous *CaM* genes (*CaM* 1*–*4 and 7–9) and 10 sequences that encoded calmodulin-like proteins (CMLs, K13448; Additional file 6).

CBL proteins fulfill crucial functions in diverse Ca^2+^-dependent processes in plants. CBL-CIPK complexes are instrumental in relaying plant responses to many environmental signals and in regulating ion fluxes [11–13, 54]. Genome analyses have revealed that 10 CBLs that specifically interact with distinct family members of the 26 CIPKs form a network-like signaling system for specific stimulus–response coupling [54, 55]. Our study finally discovered four unigene sequences that encode CBLs and 11 sequences that encode CIPKs (Additional file 6).

Among several known classes of Ca^2+^ binding sensory proteins, CDPKs are the best characterized and they are particularly important. Currently, most of the known calcium-stimulated protein kinase activities in plants are associated with CDPKs. Analysis of the genome sequence of *Arabidopsis* has indicated the presence of 34 CDPKs [56]. Based on sequence similarities, a total of 18 unigenes ranging from 151 bp to 1536 bp in the RNA-seq dataset were identified as CDPKs, including CDPK1, 2, 5, 6, 9, 11, 19 and 23 (Additional file 6).

### Validation and expression analysis of genes involved in calcium-dependent signal transduction pathways

Full-length cDNA sequences of four selected genes from *CBLs* in Ca^2+^ dependent signal transduction pathways were isolated by T-A cloning, and compared with the unique sequences to check the quality of the annotation data from Solexa sequencing. The full cDNA length of these genes varied from 639 bp to 801 bp and the DNA length ranged from 1,788 bp to 2,969 bp. Overall, the assembled unique sequences covered more than 98 % of the corresponding genome and the sequence variation was minimal (>98 % pairwise identity), which confirmed that the RNA-seq based procedures of next generation sequencing were reliable (Table 3).

**Table 3:** Sequence analyses of the four pear genes involved in Ca^2+^-dependent signal transduction pathways

Gene	Full-length cDNA	Full-length DNA	Coverage	ORF similarity
*PdCBL1*	642	2,969	99.53 %	100 %
*PdCBL2*	681	1,927	98.09 %	98.13 %
*PdCBL7*	639	1,788	98.13 %	99.39 %
*PdCBL10*	801	1,983	98.60 %	99.33 %

**Fig. 7:**
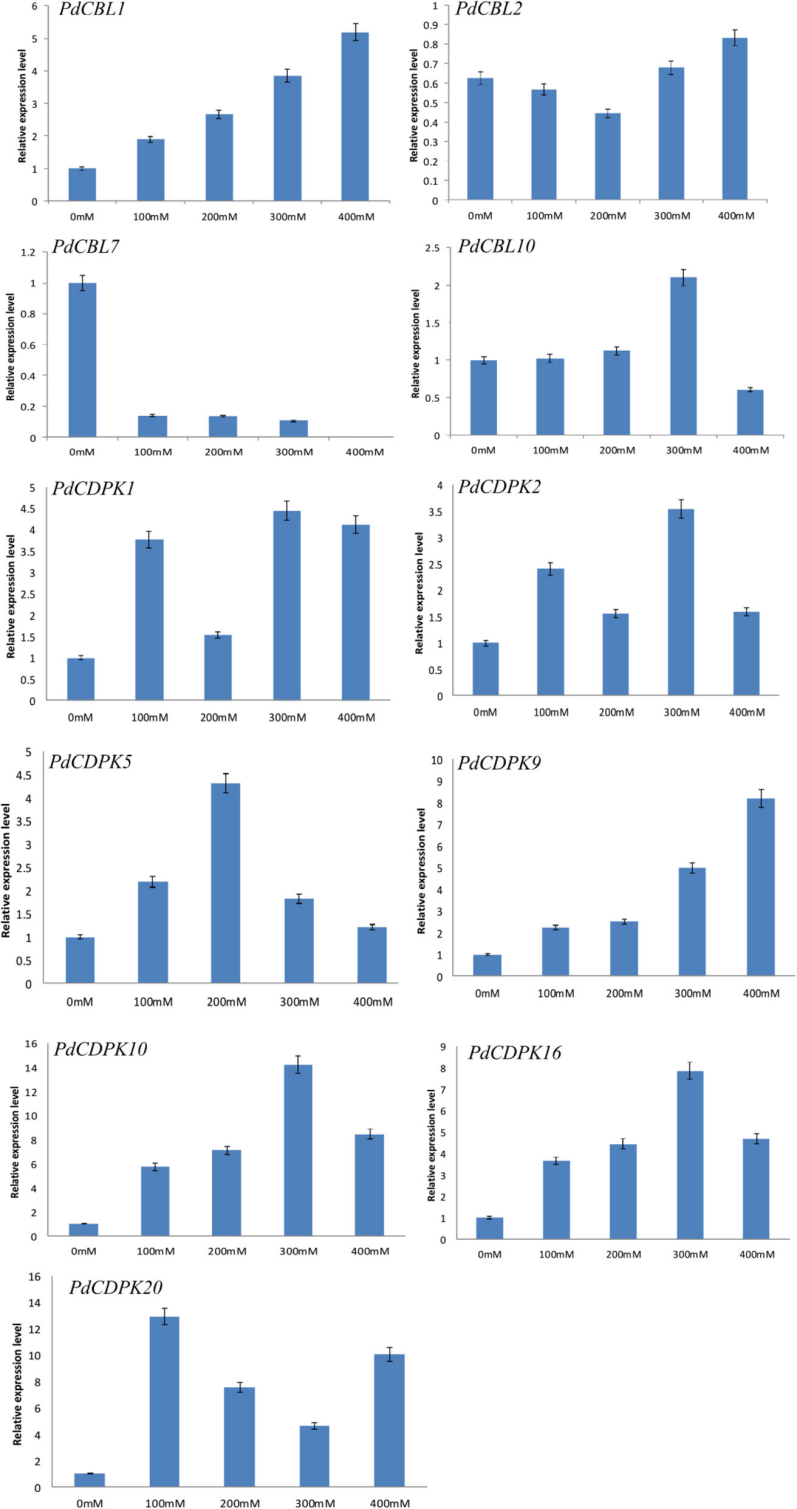
RT-qPCR expression analyses of 11 selected gene expression levels under different NaCl treatments. X axes indicate NaCl concentration. Y axes indicate relative expression level of each selected gene. Different letters indicate significant differences at *P* < 0.05 according to Duncan’s multiple range tests

To further investigate the expression of genes involved in Ca^2+^-dependent signal transduction pathways, RT-qPCR analysis was performed on eight selected genes, including four *CBL* genes (*PdCBL1*, *2*, *7* and *10*) and six *CDPK* genes (*PdCDPK1*, *2*, *5*, *9*, *10*, *16* and *20*), to provide detailed analysis of the effects of different concentrations of NaCl (0, 100, 200, 300 and 400 mM) after treatment for 6 h. Several genes involved in calcium signaling pathways showed distinct patterns of expression in different species undergoing salt stress, such as *CBL* genes in *A. thaliana* and *Populus* [57–59], and *CDPK* genes in *A. thaliana*, *Oryza sativa*, *Triticum aestivum*, *Nicotiana tabacum* and *Glycine max* [60–64]. The expression of all these genes in pear leaves varied with NaCl concentration (Fig. 7). Levels of *PdCBL7* declined gradually as the strength of NaCl treatment increased. Expressions of the remaining genes were all up-regulated compared to the control, except that *PdCBL2* showed down-regulated in response to NaCl exposure for 100 and 200 mM and *PdCBL10* was down-regulated at 400 mM.

## Conclusions

In this study, NGS-based RNA sequencing for transcriptome methods (RNA-seq) was employed to characterize the responses of pear plants exposed to salt stress. A total of 78,695 unigenes were annotated successfully by BLASTX analysis using several publicly available protein databases. The main genes that were activated following the exposure of pear leaves to salt were predominately involved in basic biological processes, biosynthesis of secondary metabolites, signal transduction mechanisms, and other cellular components and molecular functions (based on their matches in the COG, GO and KEGG databases). Additionally, 64.58 % of the unique transcripts were sequenced and successfully mapped to the pear genome, and 45.39 % were mapped to the *de novo* assembled results of pear. Furthermore, a total of 2,855 novel transcripts, 218,167 SNPs and 23,248 indels were identified successfully, and 18,322 alternative splicing events occurred. These findings demonstrated that the RNA-seq based technology is a rapid and cost-effective method for novel gene discovery in pear plants. The coverage of pear unigenes was comprehensive enough to allow for the discovery of almost all genes known to be involved in calcium signaling transduction and related pathways. The transcriptome dataset generated by this study will facilitate the understanding of molecular mechanisms via the regulation of calcium signaling. It will also provide experimental data useful for the development of salinity resistance in pear breeding programs.

## Methods

### Plant materials and RNA extraction

This study used seedlings of Callery Pear (*P. calleryana* ‘Decne’) growing at the National Pear Germplasm Repository in the Institute of Horticulture, Jiangsu Academy of Agricultural Sciences, Nanjing, China. Seeds that had been surface-sterilized and germinated were sown in a greenhouse maintained at 25 °C for 14 h in light and 18 °C for 10 h in the dark. For transcriptome sequencing, seedlings at the 4-leaf stage were transferred from soil to 1/2 MS solution (containing 200 mM NaCl) and treated for 72 h. Seedlings transferred to 1/2 MS solution without NaCl were used as a control. Leaves were collected from the control and NaCl treated plants at the same time. For qRT-PCR verification, another set of seedlings at the 4-leaf stage was transferred to 1/2 MS nutrient solution containing 0, 100, 200, 300 and 400 mM NaCl, and the plants were exposed to their respective salt treatments for 6 h. Leaves of all treated plants were collected at the same time. All samples were collected in triplicate, frozen in liquid nitrogen, and stored at −80 °C until their use for total RNA extraction. Total RNA from the seedlings was isolated using the Spectrum Plant Total RNA Kit according to the manufacturer’s protocol (Sigma-Aldrich, USA). RNase-free DNase I (Takara, Japan) was used to avoid DNA contamination. RNA samples were then quantified and examined for protein contamination (A260/A280) and reagent contamination (A260/A230) using a NanoDrop ND-1000 spectrophotometer.

### cDNA library preparation and Illumina sequencing

Poly (A) mRNA was enriched from 20 μg of total RNA from the NaCl-treated pear samples, using Sera-mag Magnetic Oligo (dT) beads (Thermo Fisher Scientific, USA). To avoid priming bias when synthesizing cDNA, the purified mRNA was fragmented to small pieces (~200 bp) using 1× fragmentation solution (Ambion, USA) at 94 °C for 5 min. These short mRNA fragments were used as templates, and random hexamer-primers were used as primers for first-strand cDNA synthesis. Double-stranded cDNA was synthesized using the SuperScript Double-Stranded cDNA Synthesis Kit (Invitrogen, USA). After synthesis of the second strand of cDNA, cDNA fragments went through an end repair and poly-A addition process, followed by ligation of sequencing adapters. The suitable fragment products were purified using agarose gel electrophoresis and enriched by PCR amplification to create a final library. The mixed cDNA library named ‘PDX’ was constructed using an mRNA-seq assay for paired-end transcriptome sequencing, which was performed by the Beijing Genomics Institute (Shenzhen, China) using an Illumina HiSeq™ 2000 platform, and 90 bp paired-end reads were generated. The sequencing data were deposited in the NCBI Sequence Read Archive (SRA, http://www.ncbi.nlm.nih.gov/sra/) as accession number SRX525736.

### Raw sequence processing and *de novo* assembly

Raw reads generated by Illumina Hiseq^TM^2000 were initially processed to get clean reads. Then, *de novo* assembly of all clean reads was performed using the program Trinity (http://trinityrnaseq.github.io/). Firstly, clean reads with a certain length of overlap were combined to form longer contiguous sequences (contigs). The reads were then mapped back to contigs using Trinity to constract unigenes with the paired-end information. The distance and relation among these contigs was calculated based on paired-end reads, which enabled the detection of contigs from the same transcript and also calculation of distances among them. Next, the contigs were further connected with Trinity, and sequences that could not be extended on either end were obtained, and defined as unigenes [50].

### Functional annotation and classification of the unigene

BLAST alignment was performed with a typical cut-off *E* value of 10^−5^ between unigenes, using publically available protein data from several databases: NCBI non-redundant protein (NR), Swiss-Prot protein, Clusters of Orthologous Groups (COGs), Gene Ontology (GO), and the Kyoto Encyclopedia of Genes and Genomes (KEGG). The best aligning outputs were used to identify sequence direction of the unigenes. If results from different databases conflicted, we followed an order of priority of NR, Swiss-Prot, KEGG and COG. ESTScan was used to decide the sequence direction and to predict coding regions of a unigene if no alignment resulted from the above databases [65]. The BLAST2GO program with NR annotation was used to get GO annotations based on biological processes, molecular functions, and cellular components ontologies [66]. Then, WEGO software was used for GO functional classification of all unigenes, to understand the distribution of gene functions at a macro level [67].

### Detection of novel transcripts, alternative splicing, SNPs and indels

To identify novel transcripts, assembled transcripts were compared to those for which reference sequences were annotated. Reference genome and gene model files were downloaded from the pear genome website at http://peargenome.njau.edu.cn/.

To be reported as a novel transcript, an assembled transcript must meet three requirements: it must be ≥ 200 bp away from the annotated gene, longer than 180 bp, and have a sequencing depth ≥ 2. To discover the functions of the novel transcripts, the Coding Potential Calculator algorithm (http://cpc.cbi.pku.edu.cn/) was used to predict whether they had the ability to encode proteins [31, 68]. We chose TopHat v1.4.0 to align paired-end clean reads to the reference genome, because this mapping tool can generate a database of splice junctions based on the gene model annotation file, thus producing a better mapping result than other non-splice mapping tools [31, 69]. Clean reads were aligned to the reference genome using the Short Oligonucleotide Analysis Package (SOAP2), and then multi-mapped reads and duplicated reads were filtered from the alignment results in order to eliminate the PCR interference and ambiguous mapping [32, 70]. Four basic types of alternative splicing were classified into exon skipping (ES), intron retention (IR), alternative 5’ splicing site (A5SS) and alternative 3’ splicing site (A3SS) [32, 71]. For intron retention, the retained regions needed to have at least ten-fold base coverage for each nucleotide position. Each accepted exon-exon junction had to be supported by at least two non-redundant reads spanning the junction. A single-nucleotide polymorphism (SNP) is a single nucleotide mutation that occurred in some individual samples based on DNA or RNA levels. SOAPsnp was used to detect SNPs; this program is a re-sequencing utility that can assemble a consensus sequence for the genome of a newly sequenced individual, based on alignment of raw sequencing reads with the known reference to obtain a consensus [70].

To detect indels, we used the Best Practices workflow for SNP and indel calling on RNAseq data available from the GATK website (https://www.broadinstitute.org/gatk/guide/topic?name=methods#methods3891). In brief, the reads were aligned to the genome of diploid *P. bretschneideri Rehd*. cv. Dangshansuli (also known as Suli) using STAR aligner. Specifically, we utilized the STAR 2-pass method, described recently [72]. Finally, the HaplotypeCaller was applied for indel calling. To ensure the accuracy of results, the results were filtered using stringent criteria (Fisher Strand values, FS > 200.0, Qual By Depth values, QD < 2.0, InbreedingCoeff < −0.8, and ReadPosRankSum < −20.0), and the filtered results were annotated by SnpEff [73].

### Gene validation by T-A cloning and sequencing

Degenerate and specific PCR primers were designed for the *CBL* genes, corresponding to the conserved amino acid sequences in the analagous gene products of other plant species (Additional file 7). PCR was performed in a total volume of 25 μL containing 2.0 mmol/L Mg^2+^, 0.15 mmol/L dNTPs, 0.4 mmol/L of each primer, 0.8 U Taq DNA polymerase (TAKARA) and 10 ng cDNA under the following conditions: an initial denaturation step at 94 °C for 3 min, 30 cycles at 94 °C for 50 s, 58 °C for 50 s, 72 °C for 100 s, with a final extension at 72 °C for 10 min followed by holding at 4 °C. The PCR products were separated by agarose gel electrophoresis and the incised gels were purified using the Agarose Gel DNA Purification Kit Ver.2.0 (TaKaRa). All products were ligated into the pMD19-T simple vector system (TaKaRa). Positive clones were sequenced with ABI3730 at Invitrogen Biotechnology Service Company (Shanghai, China).

### Quantitative real-time PCR analysis

Quantitative real-time PCR was carried out in 96-well plates with an ABI7500 Real-Time PCR Detection System (Applied Biosystems, USA) using SYBR Green Master ROX (Roche, Japan) following the manufacturer’s instructions. Primers were designed using Beacon Designer 7.0 (Premier Biosoft International, USA); Primer 5.0 (Premier Biosoft International, CA) and the reference genes *ACT2/7* and *UBQ10* were selected for use based on previous research (Additional file 8). Reaction mixtures contained 10 μL SYBR Green Mix, 0.2 μM of each primer and 1.5 μL of 1:10 diluted cDNA as template in a final volume of 20 μL. Reactions were subjected to the following conditions: 95 °C for 10 min, followed by 40 cycles of 95 °C for 30 s and 58 °C for 1 min [26, 74]. After the reactions, a melting curve analysis was conducted to evaluate the primer specificity. The relative expression levels of the selected transcripts, normalized to *ACT2/7* and *UBQ10*, were calculated using the 2^-∆∆Ct^ method. All reactions were performed with three independent biological replicates, and the expression levels calculated for each sample were based on three technical replicates. Data were analyzed using the ABI7500 manager software, and statistical analyses were conducted with SAS Version 9.0 software (SAS Institute, Cary, NC, USA) using Duncan’s multiple range test at the *P* < 0.05 level of significance.

## Additional files

Additional file 1:
**Length distribution of the coding sequence (CDS) and predicted proteins by BLASTX and ESTScan software from the unique sequences.** A: Aligned CDS by BLASTX. B: Proteins predicted by BLASTX. C: Aligned CDS by ESTScan. D: Proteins predicted by ESTScan. (DOC 2301 kb) Additional file 2:
**KEGG pathways of the unique sequences.** (XLS 712 kb) Additional file 3:
**Identification and classification of novel transcripts based on the number of exons.** (DOC 40 kb) Additional file 4:
**Summary statistics for indel analysis.** (DOC 39 kb) Additional file 5:
**Candidate genes involved in MYB transcript factors.** (XLS 286 kb) Additional file 6:
**Candidate genes involved in calcium signaling pathways in pear plants.** (XLS 98 kb) Additional file 7:
**Primers used for T-A cloning and sequencing.** (DOC 33 kb) Additional file 8:
**Primers used for RT-qPCR.** (DOC 36 kb)
