# Supplementary material for: Transcriptome sequencing and analysis of major genes involved in calcium signaling pathways in pear plants (Pyrus calleryana Decne.)
Source: BMC Genomics. 2015 Sep 30;16:738. doi: 10.1186/s12864-015-1887-4 (PMC4590731; doi:10.1186/s12864-015-1887-4)
Supplement: Additional file 1: — Length distribution of the coding sequence (CDS) and predicted proteins by BLASTX and ESTScan software from the unique sequences. A: Aligned CDS by BLASTX. B: Proteins predicted by BLASTX. C: Aligned CDS by ESTScan. D: Proteins predicted by ESTScan. (DOC 2301 kb) [file 12864_2015_1887_MOESM1_ESM.doc]

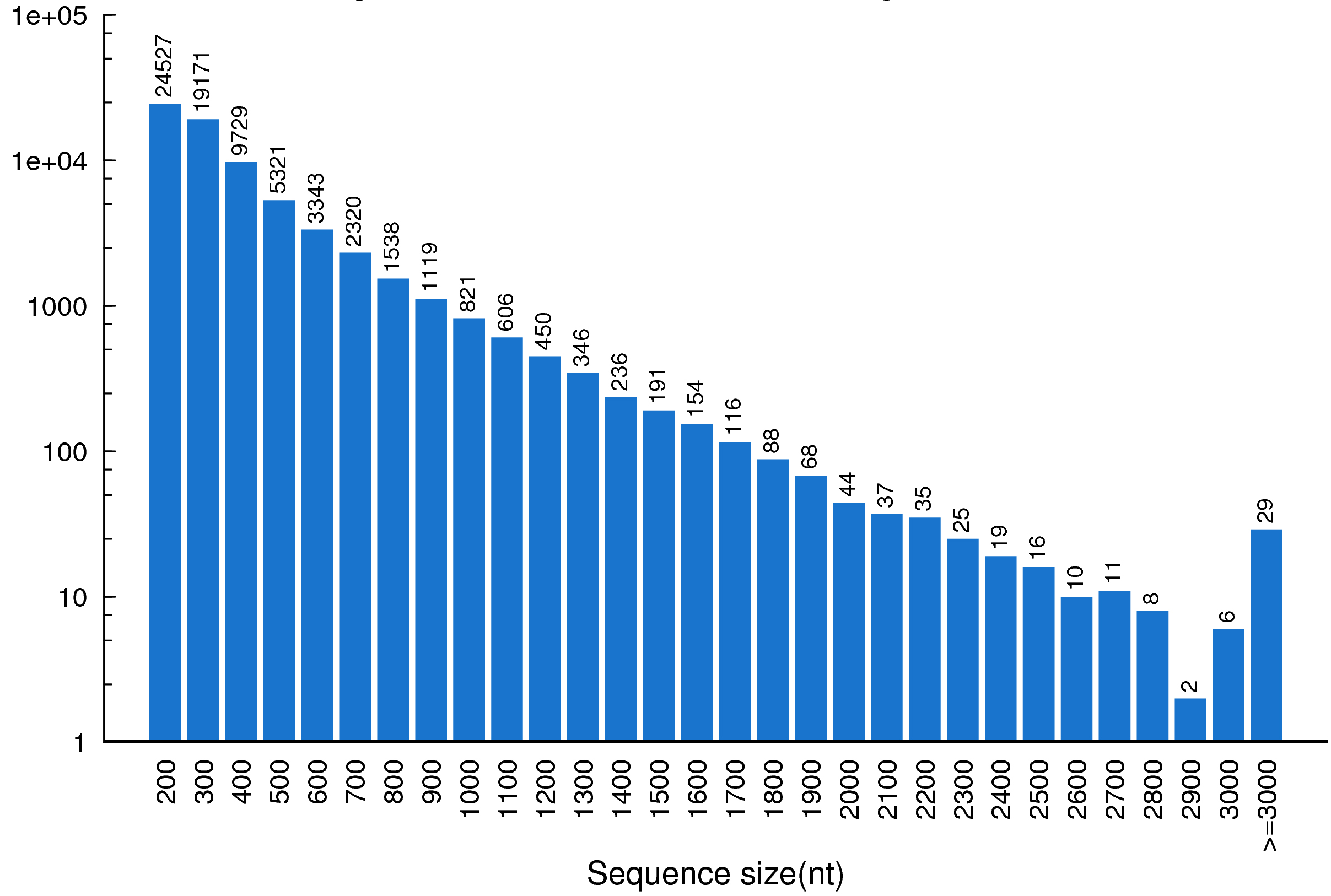


**A**

**Number of aligned CDS**


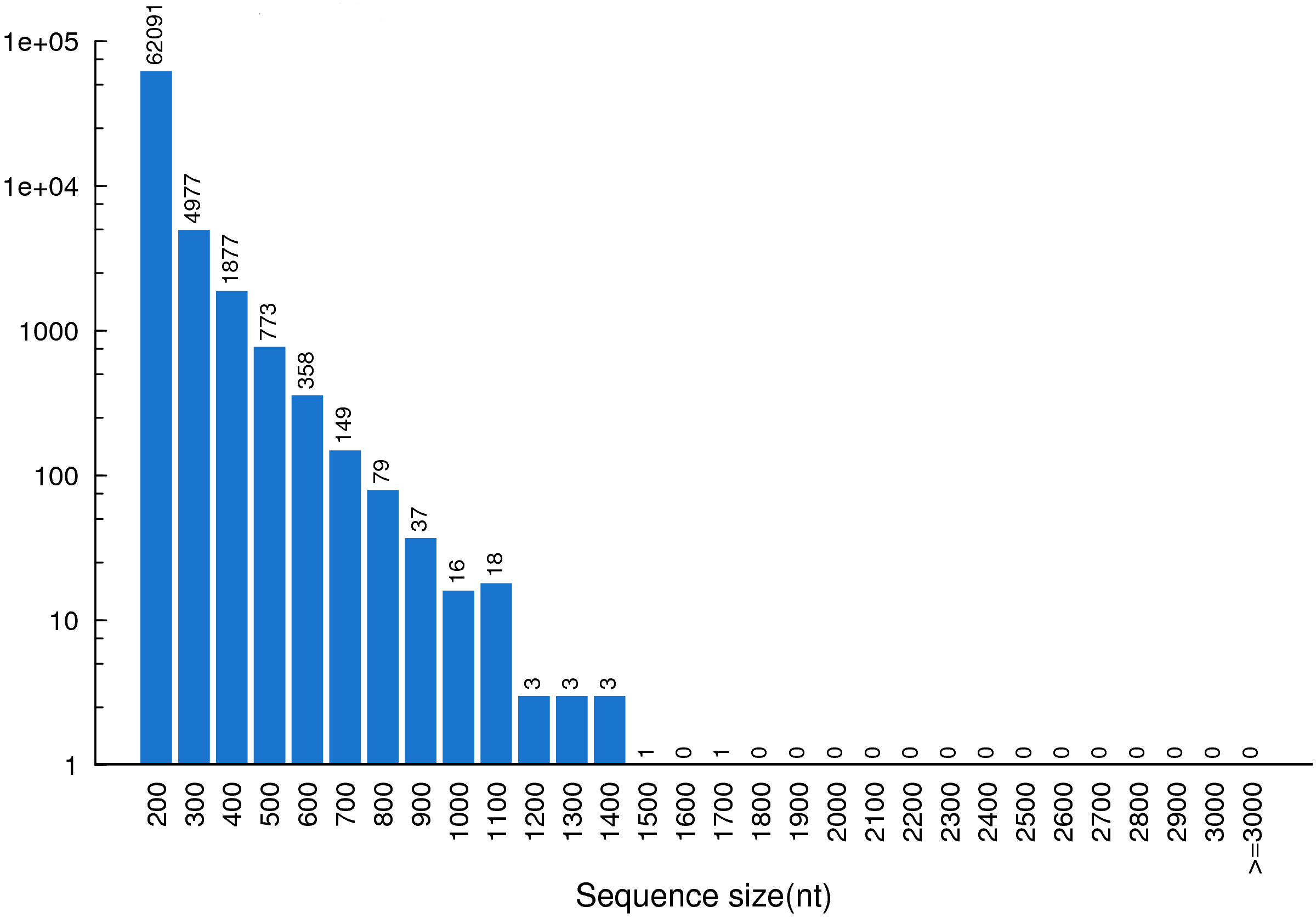


**B**

**Number of predicted protein**


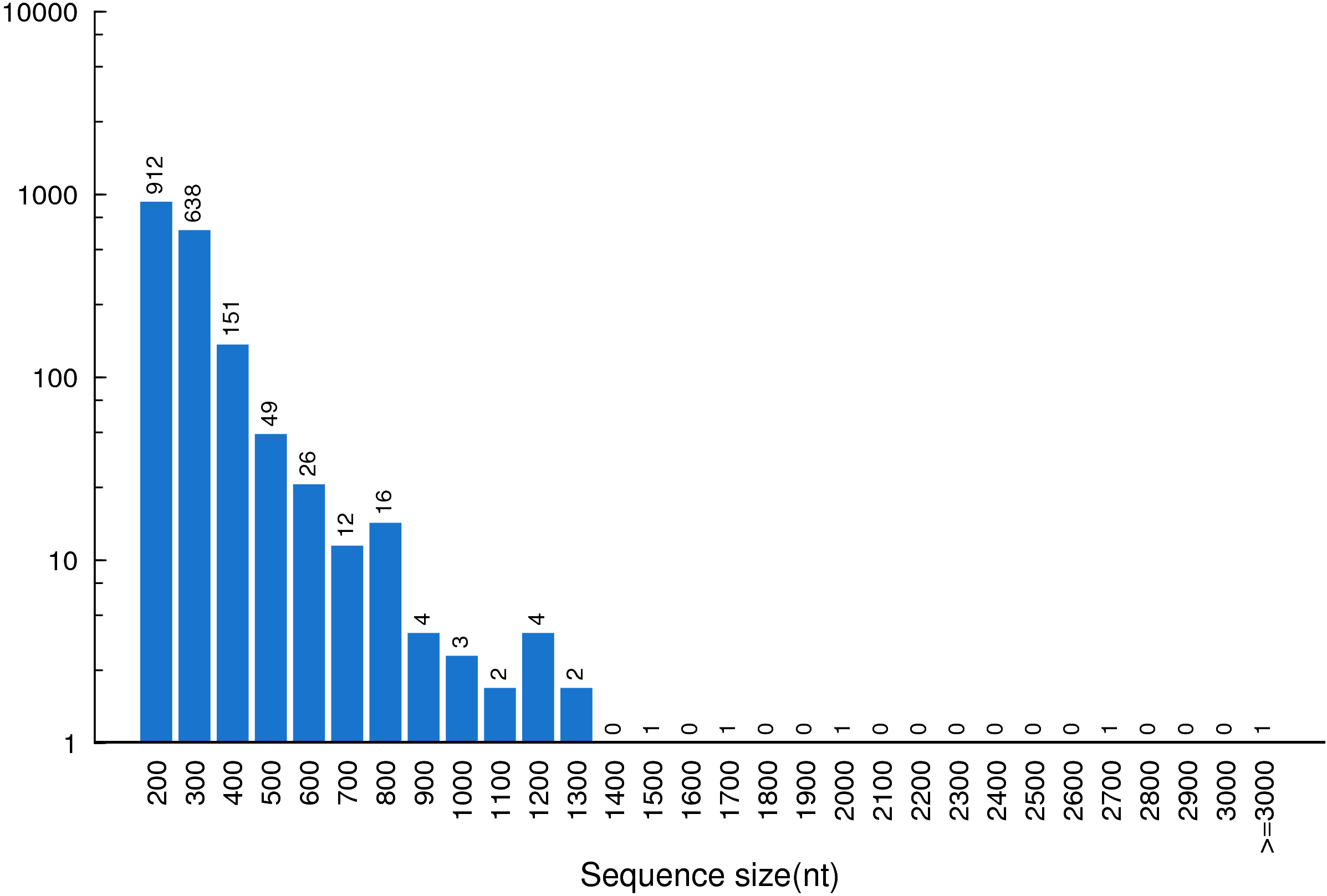


**C**

**Number of aligned CDS**


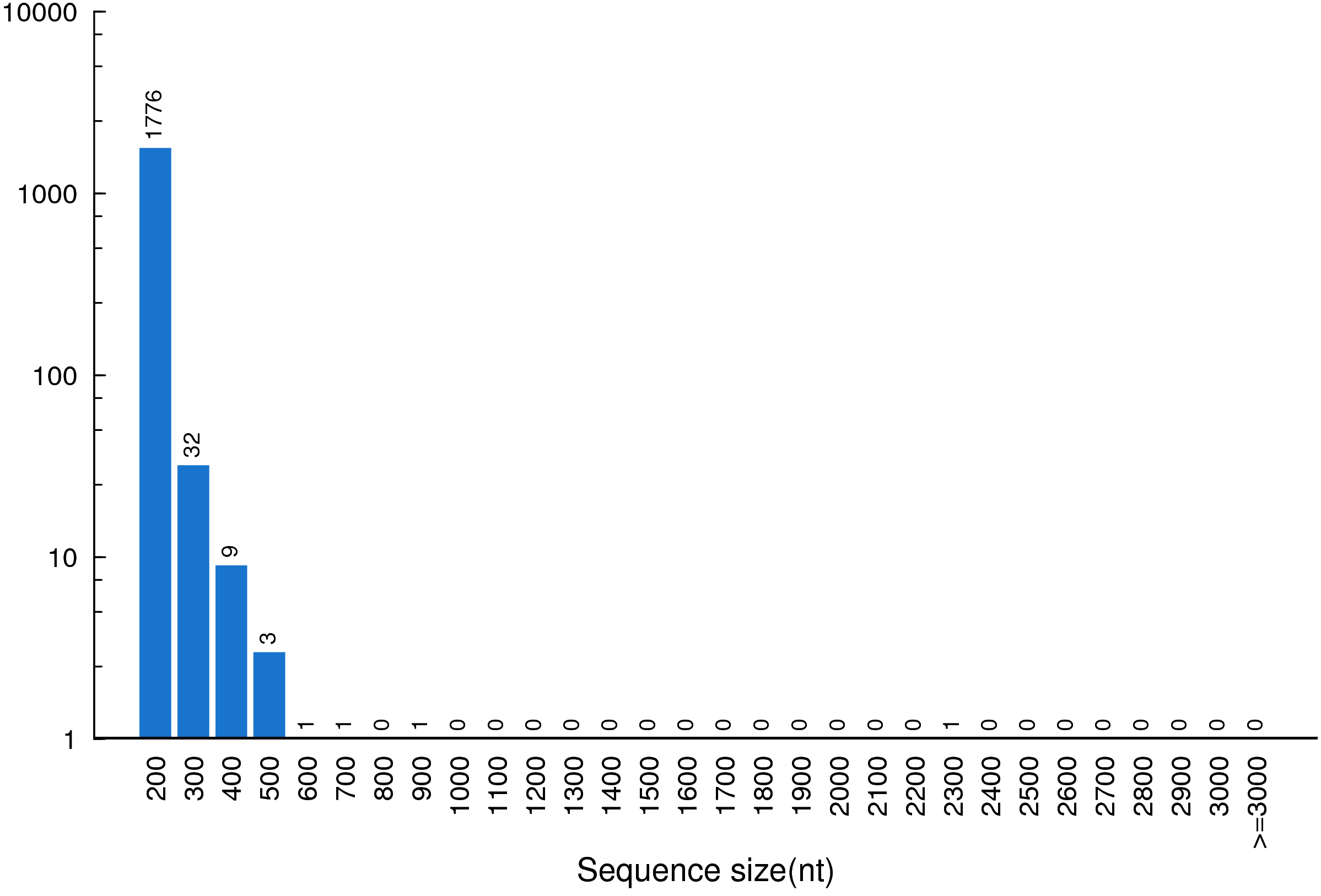


**Number of predicted protein**

**D**

**Additional file 1:** **Length distribution of the coding sequence (CDS) and predicted proteins by BLASTX and ESTScan software from the unique sequences.** A: Aligned CDS by BLASTX. B: Proteins Predicted by BLASTX. C: Aligned CDS by ESTScan. D: Proteins predicted by ESTScan.
