# Supplementary material for: Transcriptome sequencing and analysis of major genes involved in calcium signaling pathways in pear plants (Pyrus calleryana Decne.)
Source: BMC Genomics. 2015 Sep 30;16:738. doi: 10.1186/s12864-015-1887-4 (PMC4590731; doi:10.1186/s12864-015-1887-4)
Supplement: Additional file 3: — Identification and classification of novel transcripts based on the number of exons. (DOC 40 kb) [file 12864_2015_1887_MOESM3_ESM.doc]

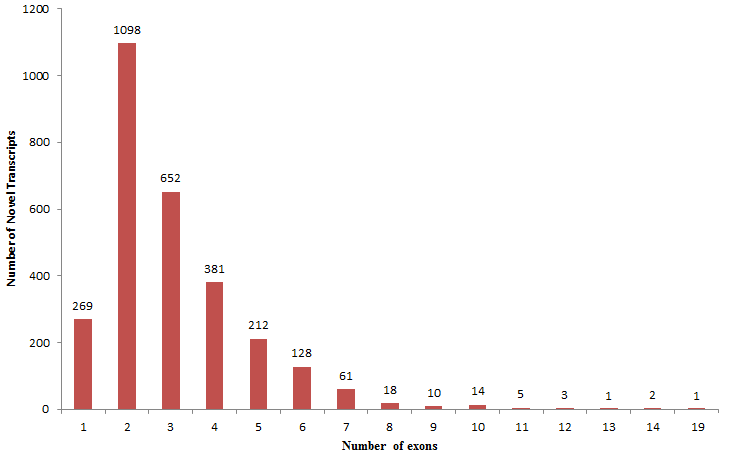


**Additional file 3** **Identification and classification of novel transcripts based on the number of exons.**
