# Supplementary material for: Transcriptome sequencing and analysis of major genes involved in calcium signaling pathways in pear plants (Pyrus calleryana Decne.)
Source: BMC Genomics. 2015 Sep 30;16:738. doi: 10.1186/s12864-015-1887-4 (PMC4590731; doi:10.1186/s12864-015-1887-4)
Supplement: Additional file 4: — Summary statistics for indel analysis. (DOC 39 kb) [file 12864_2015_1887_MOESM4_ESM.doc]

**Additional file 4 Summary statistics of indel analysis.**

|  | Statistical results |
| --- | --- |
| Total | 23,248 |
| Frameshift Insertion | 1,503 |
| Non-frameshift Insertion | 1,196 |
| Frameshift Deletion | 1,817 |
| Non-frameshift Deletion | 1,594 |
| Stopgain | 6 |
| Stoploss | 5 |
| Intronic | 2,269 |
| Upstream | 6,156 |
| Downstream | 3,344 |
| Intergenic | 536 |
| UTR5 | 2,465 |
| UTR3 | 2,353 |
| Hom | 3,864 |
| Het | 19,384 |
| Splice | 214 |
| Ins/del | 0.805 |
| Insertion | 10,366 |
| Deletion | 12,881 |
| Replacement | 1 |
