# Supplementary material for: Transcriptome sequencing and analysis of major genes involved in calcium signaling pathways in pear plants (Pyrus calleryana Decne.)
Source: BMC Genomics. 2015 Sep 30;16:738. doi: 10.1186/s12864-015-1887-4 (PMC4590731; doi:10.1186/s12864-015-1887-4)
Supplement: Additional file 7: — Primers used for T-A cloning and sequencing. (DOC 33 kb) [file 12864_2015_1887_MOESM7_ESM.doc]

**Additional file 7 Primers used for T-A cloning and sequencing.**

| Gene | Forward primers (5′-3′) | Reverse primers (5′-3′) |
| --- | --- | --- |
| *PdCBL1* | TAGATGCTCTAAATGGGTTG | GTCATGTAGCAATCTCAT |
| *PdCBL2* | AGAAAATATCATGTTGCAG | TCAGGTGTCGTCAACTTG |
| *PdCBL7* | GTTTGTTAATGGGCTGCTAT | AATCACATTTCTGAATCCT |
| *PdCBL10* | CGCCGATGGCTGAGAGAC | TCAGTCTTCGACTCCAGT |
