# Supplementary material for: Transcriptome sequencing and analysis of major genes involved in calcium signaling pathways in pear plants (Pyrus calleryana Decne.)
Source: BMC Genomics. 2015 Sep 30;16:738. doi: 10.1186/s12864-015-1887-4 (PMC4590731; doi:10.1186/s12864-015-1887-4)
Supplement: Additional file 8: — Primers used for RT-qPCR. (DOC 36 kb) [file 12864_2015_1887_MOESM8_ESM.doc]

**Additional file 8 Primers used for RT-qPCR.**

| Gene | Forward primers (5′-3′) | Reverse primers (5′-3′) |
| --- | --- | --- |
| *PdCBL1* | AAGATAGACAAGTATGAATGGAAC | TGTAGCAATCTCATCAACCT |
| *PdCBL2* | CAAGGAAGAGTGGAGAAG | GAATGTTGTGGTGATGTC |
| *PdCBL7* | ATTAGGCGTCTTTCACCC | GACCACCATCTCCTTCAACT |
| *PdCBL10* | ATCGGCGAGACGCTCTGT | TCGTGGGTGAAGGAAAGG |
| *PdCDPK1* | GTGTTGGACCGAGCATTT | ACCGAACCAGCCTGAAGT |
| *PdCDPK2* | AACTGCTGCTCAGGCTCT | TTGCTGTCAATCGCTTCC |
| *PdCDPK5* | GCCGATGTCTGGAGTGTA | TCCGTGGGTCTTTATTCA |
| *PdCDPK9* | ATTCGGCGTGACCTATCT | CCCTCTTGATGTCCTCCT |
| *PdCDPK10* | TGGAACCGATTGGAGAAA | CAGTTAGCCCGTCGTGAA |
| *PdCDPK16* | TCCACCTATCTGCTCCAA | TTATGAACAGCCTGCCTC |
| *PdCDPK20* | GCCATTCTTTGGGTGTCA | ACCTTGCTCCGTTTCATC |
| *ACT2/7* | CTCCCAGGGCTGTGTTTCCTA | CTCCATGTCATCCCAGTTGCT |
| *UBQ10* | TAAGCAGTTGGAGGATGGAA | ACGGAGGACAAGGTGAAG |
